# Generating Porcine Chimeras Using Inner Cell Mass Cells and Parthenogenetic Preimplantation Embryos

**DOI:** 10.1371/journal.pone.0061900

**Published:** 2013-04-23

**Authors:** Kazuaki Nakano, Masahito Watanabe, Hitomi Matsunari, Taisuke Matsuda, Kasumi Honda, Miki Maehara, Takahiro Kanai, Gota Hayashida, Mirina Kobayashi, Momoko Kuramoto, Yoshikazu Arai, Kazuhiro Umeyama, Shuh-hei Fujishiro, Yoshihisa Mizukami, Masaki Nagaya, Yutaka Hanazono, Hiroshi Nagashima

**Affiliations:** 1 Laboratory of Developmental Engineering, Department of Life Sciences, School of Agriculture, Meiji University, Kawasaki, Japan; 2 Meiji University International Institute for Bio-Resource Research (MUIIBR), Kawasaki, Japan; 3 Division of Regenerative Medicine, Center for Molecular Medicine, Jichi Medical University, Tochigi, Japan; 4 CREST, Japan Science and Technology Agency, Tokyo, Japan; National University of Singapore, Singapore

## Abstract

**Background:**

The development and validation of stem cell therapies using induced pluripotent stem (iPS) cells can be optimized through translational research using pigs as large animal models, because pigs have the closest characteristics to humans among non-primate animals. As the recent investigations have been heading for establishment of the human iPS cells with naïve type characteristics, it is an indispensable challenge to develop naïve type porcine iPS cells. The pluripotency of the porcine iPS cells can be evaluated using their abilities to form chimeras. Here, we describe a simple aggregation method using parthenogenetic host embryos that offers a reliable and effective means of determining the chimera formation ability of pluripotent porcine cells.

**Methodology/Significant Principal Findings:**

In this study, we show that a high yield of chimeric blastocysts can be achieved by aggregating the inner cell mass (ICM) from porcine blastocysts with parthenogenetic porcine embryos. ICMs cultured with morulae or 4–8 cell-stage parthenogenetic embryos derived from *in vitro*-matured (IVM) oocytes can aggregate to form chimeric blastocysts that can develop into chimeric fetuses after transfer. The rate of production of chimeric blastocysts after aggregation with host morulae (20/24, 83.3%) was similar to that after the injection of ICMs into morulae (24/29, 82.8%). We also found that 4–8 cell-stage embryos could be used; chimeric blastocysts were produced with a similar efficiency (17/26, 65.4%). After transfer into recipients, these blastocysts yielded chimeric fetuses at frequencies of 36.0% and 13.6%, respectively.

**Conclusion/Significance:**

Our findings indicate that the aggregation method using parthenogenetic morulae or 4–8 cell-stage embryos offers a highly reproducible approach for producing chimeric fetuses from porcine pluripotent cells. This method provides a practical and highly accurate system for evaluating pluripotency of undifferentiated cells, such as iPS cells, based on their ability to form chimeras.

## Introduction

In recent years, dramatic progress has been made in research into the application of induced pluripotent stem (iPS) cells for the treatment of both intractable genetic diseases and acquired disorders such as myocardial infarction [1], macular degeneration [2], [3] and spinal injury [4]. However, before iPS cells can be used for clinical therapies, extensive animal experiments are necessary to elucidate therapeutic mechanisms and to evaluate the efficacy and safety of potential treatments. Although a range of experimental animal models is available, it is preferable that such investigations are conducted on those with anatomical and physiological similarities to humans. For this reason, pigs are often chosen for use in translational research because they possess the closest similarity to humans among non-primate animal models [5], [6], and consequently, the results of research on porcine iPS cell therapy [7], [8] can be more easily extrapolated to humans.

Recent investigations have been heading for establishment of human iPS cell with naïve state characteristics [9]–[11]. Similarly, development of the naïve type porcine iPS cells [12] has become an important challenge in translational research using pigs, since the pioneering works on porcine iPS cells [7], [12]–[16]. Thus, the pluripotency of porcine iPS cells need to be evaluated by a reliable means. Authentic pluripotency of the iPS cells can be proven by their competence to contribute to chimera formation. To date, however, the ability of porcine iPS cells to form chimeras is still very limited. In general, the methods used to judge whether established pluripotent cells possess the ability to form chimeras evaluate the success of incorporation of sample cells (donor cells) into developing host embryos and their contribution to the fetus or offspring. This is a lengthy and involved test procedure and has stimulated a search for more simple and efficient methods for proving pluripotency.

The incorporation of donor cells into a host embryo can be achieved either by injecting donor cells directly into the blastocoele of a host blastocyst [17] or by an aggregation method in which donor cells are co-cultured with the host embryo [18], [19]. Although the former method has been used successfully to show the ability of donor cells to form chimeras [12], [15], [20]–[26], the method requires a high skill level and expensive equipments. In contrast, the aggregation method offers a simple approach to generating chimeric embryos [27].

There have been a number of advances in pig reproductive technologies in recent years, including *in vitro* oocyte maturation, parthenogenetic oocyte activation, and *in vitro* embryo culture [28]–[30]. These developments have made it possible to culture parthenogenetic embryos to the somite stage [31]–[33]. Consequently, it is now feasible to use a parthenogenetic embryo as the host for the evaluation of the ability of pluripotent cells to form chimeric fetuses. The use of parthenogenetic embryos derived from *in vitro*-matured (IVM) oocytes offers the additional advantage of saving labor and costs compared to the use of *in vivo*-derived embryos.

In the present study, we demonstrate that the aggregation method can be used with parthenogenetic porcine embryos derived from IVM oocytes to efficiently generate chimeric embryos and fetuses with pluripotent cells. This system provides a simple and reliable means for evaluating the pluripotency of established iPS cell lines through comparison of their abilities to form chimeric embryos.

## Methods

### Animal Care

The pigs used in the present study were maintained in a semiwindowless facility with a controlled temperature (15–30°C) and received a standard pig diet twice a day and water *ad libitum*. All of the animal experiments in the present study were approved by the Institutional Animal Care and Use Committee of Meiji University (IACUC-11-1).

### Chemicals

Chemicals were purchased from the Sigma Chemical Co. (St. Louis, MO, USA), unless otherwise indicated.

### In vitro Oocyte Maturation

IVM oocytes were prepared as described elsewhere [34]. Pig ovaries were collected at a local abattoir and transported to the laboratory in Dulbecco’s phosphate-buffered saline (DPBS; Nissui Pharmaceutical Co., Ltd., Tokyo, Japan) containing 75 µg/ml potassium penicillin G, 50 µg/ml streptomycin sulfate, 2.5 µg/ml amphotericin B and 0.1% (w/v) polyvinyl alcohol (PVA). Cumulus-oocyte complexes (COCs) were collected by aspiration from ovarian antral follicles that had a diameter of 3.0–6.0 mm. COCs with at least three layers of compacted cumulus cells were selected and culture in NCSU23 medium [35] supplemented with 0.6 mM cysteine, 10 ng/ml epidermal growth factor, 10% (v/v) porcine follicular fluid, 75 µg/ml potassium penicillin G, 50 µg/ml streptomycin sulfate, 10 IU/ml eCG (ASKA Pharmaceutical Co., Ltd., Tokyo, Japan) and 10 IU/ml hCG (ASKA Pharmaceutical). The COCs were cultured for 22 hr with eCG and hCG in a humidified atmosphere of 5% CO_2_ and 95% air at 38.5°C. The COCs were then cultured for 22 hr without eCG and hCG in an atmosphere of 5% CO_2_, 5% O_2_ and 90% N_2_ [36]. IVM oocytes with expanded cummulus cells were treated with 1 mg/ml hyaluronidase dissolved in Tyrode’s lactose medium containing 10 mM Hepes and 0.3% (w/v) polyvinylpyrrolidone (Hepes-TL-PVP) and were separated from the cumulus cells by gentle pipetting. Oocytes with an evenly granulated ooplasm and an extruded first polar body were selected for subsequent experiments.

### Parthenogenetic Activation of Oocytes

Parthenogenetic embryos developed from IVM oocytes were used as host embryos in all the experiments. Parthenogenesis of oocytes was induced by electric activation as reported previously [34]. The oocytes were washed twice in an activation solution composed of 280 mM mannitol (Nacalai Tesque, Inc., Kyoto, Japan), 0.05 mM CaCl_2_, 0.1 mM MgSO_4_ and 0.01% (w/v) PVA. They were then aligned between two wire electrodes (1.0 mm apart) in a drop of the activation solution on a fusion chamber slide (CUY500G1, Nepa Gene, Chiba, Japan). A single direct current pulse of 150 V/mm was applied for 100 µsec using an electrical pulsing machine (LF201; Nepa Gene). Activated oocytes were treated with 5 µg/ml cytochalasin B for 3 hr to suppress extrusion of the second polar body.

### In vitro Fertilization of Oocytes

Donor embryos for producing chimeric fetuses were prepared by *in vitro* fertilization of oocytes using frozen sperm of a transgenic boar carrying humanized Kusabira-Orange (huKO) gene. *In vitro* fertilization was carried out as described elsewhere [37]. Briefly, frozen epididymal sperm [38] recovered from a straw were suspended in 5 ml DPBS supplemented with 0.1% BSA (306–1138, Wako Pure Chemical industries, Ltd., Osaka, Japan) and washed three times by centrifugation at 1,000×g for 4 min. After washing, the sperm pellets were resuspended in porcine fertilization medium (PFM) [39] (Research Institute for the Functional Peptides, Yamagata, Japan) at a concentration of 1×10^7^ cells/ml. For insemination, 20 COCs that had been matured *in vitro* were placed in a 100-µl drop of PFM containing spermatozoa (1.75×10^6^ cells/ml); the oocytes and sperm were incubated for 8 hr at 38.5°C in a humidified atmosphere containing 5% CO_2_, 5% O_2_, and 90% N_2_. After insemination, the eggs were transferred to Hepes-TL-PVP; cumulus cells and excess sperm were removed by gentle pipetting. Eggs that showed release of polar bodies with normal cytoplasmic morphology were selected for use in later experiments.

### In vitro Culture of Embryos

*In vitro* culture of the parthenogenetic and *in vitro*-fertilized (IVF) embryos was performed in droplets of porcine zygote mediume-5 (PZM-5) (Research Institute for Functional Peptides) under paraffin oil (32033-00, Kanto Chemical Co., Inc., Tokyo, Japan) in plastic 35-mm dish maintained in a humidified atmosphere of 5% O_2_ and 90% N_2_ at 38.5°C. The culture media were supplemented with 10% (v/v) fetal calf serum for culturing embryos at the morula stage or later.

### Isolation of Donor ICMs from Blastocysts

Donor ICMs were isolated from the parthenogenetic and IVF blastocysts by immunosurgery as described previously [22]. Briefly, day-6 blastocysts were incubated at 38.5°C for 15 min with heat-inactivated rabbit anti-pig spleen cell serum (BioTools Inc., Gunma, Japan) diluted 1∶8 with 21 mM Hepes-buffered Minimal Essential Medium with Earle salts, L-glutamine and nonessential amino acids (Gibco-Invitrogen, Carlsbad, CA, USA) supplemented with 5 mg/ml BSA (MEM-Hepes). Embryos were then incubated with guinea pig complement serum, diluted 1∶8 with MEM-Hepes at 38.5°C for 10–15 min. Swollen trophectodermal cells were dissociated by vigorous pipetting (inner diameter, 100 µm). The ICM was washed thoroughly with MEM-Hepes.

### Isolation of Donor Blastomeres from Parthenogenetic Embryos

Blastomeres isolated from parthenogentic embryos were also used as donor cells to produce chimeric blastocysts. The parthenogenetic embryos at the 4–8 cell (day 3) or morula (day 4) stage were decompacted by incubation in Ca^2+^/Mg^2+^-free DPBS containing 0.1 mM EDTA-2Na and 0.01% (w/v) PVA for 15–20 min, followed by removal of the zona pellucidae by digesting with 0.25% (w/v) pronase (in DPBS). Blastomeres were isolated from the zona removed embryos by gentle pipetting with a finely drawn glass capillary.

### Staining of Donor Cells for in vitro Tracing

Donor-ICMs and donor-blastomeres isolated from parthenogenetic embryos were labeled with fluorescent carbocyanine dye (DiI) (Takara Bio, Inc., Shiga, Japan) for tracing during the formation of chimeric blastocysts *in vitro*. Staining of the cells was performed according to the manufacturer’s protocol. ICMs and blastomeres were placed in MEM-Hepes containing 1% (v/v) DiI for 30 min, after which excess DiI was washed out by immersing the cells twice in MEM-Hepes for 10 min each time.

### Preparation of Chimeric Embryos

**Figure 1:**
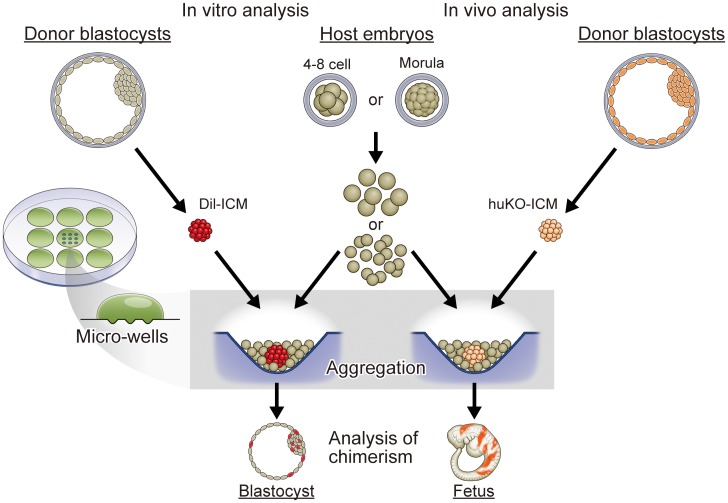
Generalized scheme for the production of chimeric porcine blastocysts and fetuses by the aggregation method. For *in vitro* analysis of the chimeric blastocyst formation, donor ICMs were isolated from parthenogenetic blastocysts derived from IVM oocytes. Isolated ICMs stained with DiI were aggregated with blastomeres isolated from parthenogenetic host embryos in a microwell made on the bottom of a culture dish. For *in vivo* analysis of chimeric fetus formation, the donor ICMs were isolated from blastocysts fertilized *in vitro* by transgenic boar sperm carrying the fluorescent huKO gene. ICMs of the IVF blastocysts were similarly aggregated with the parthenogenetic host embryos as the DiI-stained ICMs, and the resultant blastocysts were transferred to recipient pigs to obtain chimeric fetuses.

Aggregation of donor cells and host embryos was carried out using the micro-well method [40]. A cluster of 9 to 12 depressions (400 µm in diameter, 300 µm in depth) was made on the bottom of a culture dish (Iwaki 1000-35, Asahi Techno Glass, Tokyo, Japan) using an aggregation needle (BLS, Ltd., Budapest, Hungary) (Figure 1). The cluster of micro-wells was overlaid with a microdrop (30 µl) of PZM-5 and covered with paraffin oil.

Blastomeres of the host embryos were isolated from parthenogenetic morulae (day 4) and 4–8 cell stage embryos (day 3) by the same way for the donor cells as described above.

**Figure 2:**
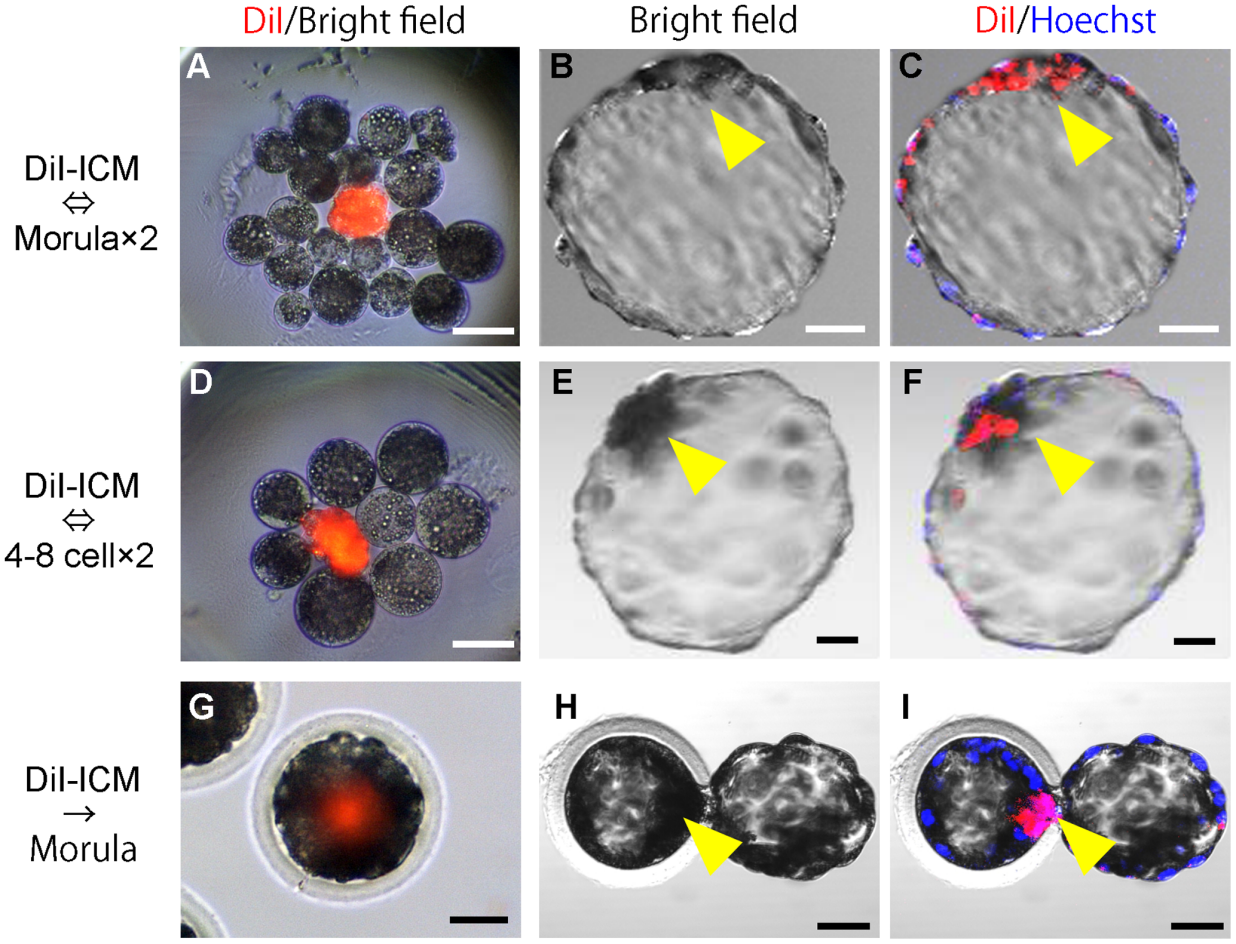
Production of chimeric blastocysts with donor ICM and parthenogenetic host embryos. (A, D) A donor ICM (stained with Dil) aggregated with host blastomeres isolated from parthenogenetic embryos at the morula (A) or 4–8 cell stage (D). (B, E) Bright field images of chimeric blastocysts developed from the aggregated embryos. (C, F) Confocal fluorescence images of chimeric blastocysts showing DiI fluorescence in ICMs. Single confocal sections of fluorescence were overlaid on the bright field images. (G-I) Parthenogenetic host morulae injected with DiI-stained donor ICM (G) and resultant chimeric blastocysts (H, I). Arrow heads, ICM. Scale bars = 50 µm.

We first examined the *in vitro* development of chimeric embryos composed of the donor ICM and host blastomeres (Figure 1). A donor ICM of parthenogenetic blastocysts was placed in each micro-well with blastomeres isolated from two host embryos (Figure 2A, D).

As a control experiment, some of the ICMs were injected into host morulae. Isolated ICMs were inserted into the center portion of the host morulae (Figure 2G) using a beveled injection pipette by micromanipulation with a micromanipulator (MO-102, Narishige, Tokyo, Japan) and injectors (IM-6, Narishige).

**Figure 3:**
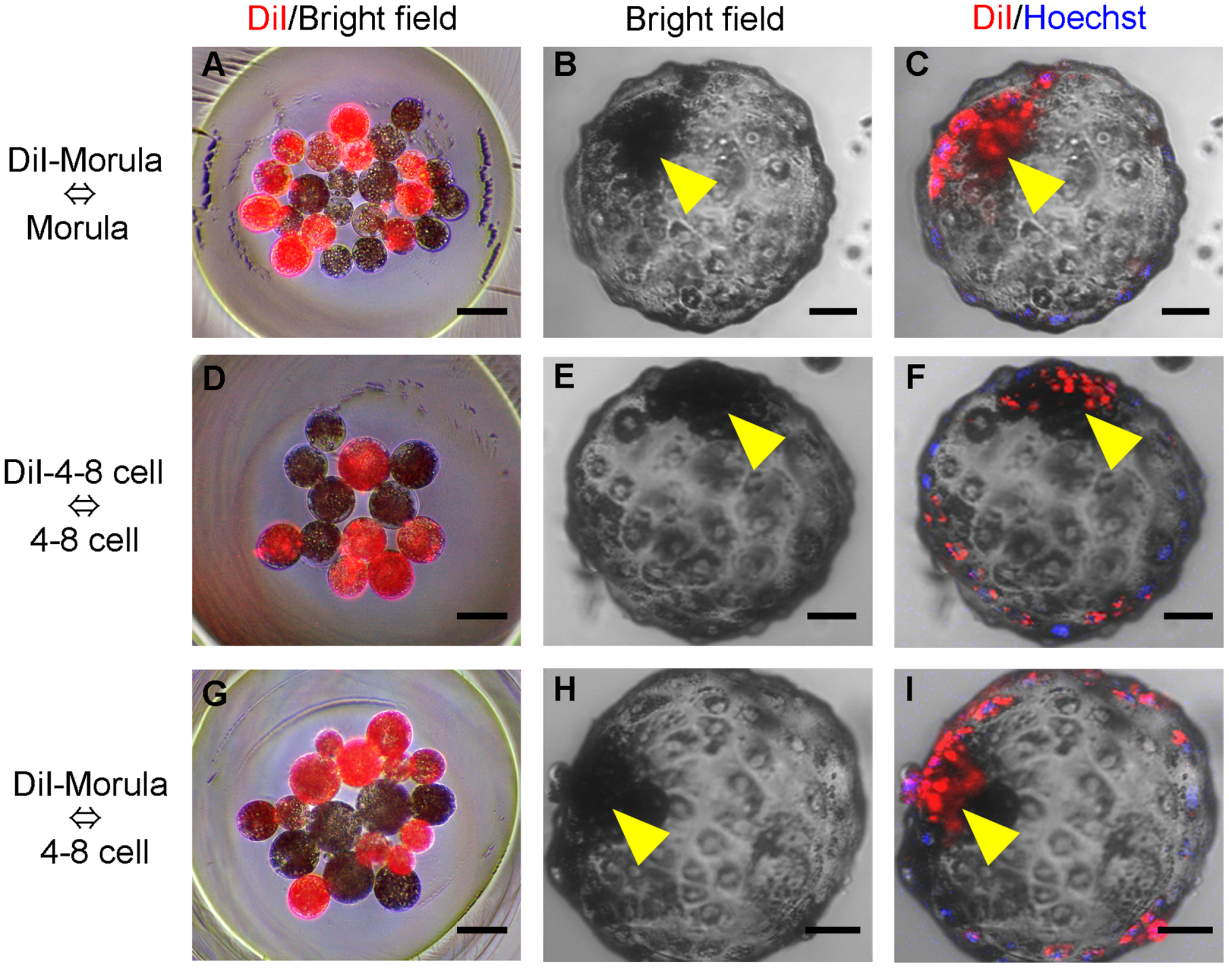
Production of chimeric blastocysts by blastomere aggregation. (A, D, G) Aggregation of donor (DiI-stained) and host blastomeres between synchronous (A, D) and asynchronous (G) embryonic stages. (B, E, H) Chimeric blastocysts developed from the aggregated blastomeres. (C, F, I) Confocal fluorescence images of the chimeric blastocysts showing DiI fluorescence in ICMs. Single confocal sections of fluorescence were overlaid on the bright field images. Arrow heads, ICM. Scale bars = 50 µm.

*In vitro* development of chimeric embryos was also analyzed using donor blastomeres instead of donor ICMs (Figure 3). Blastomeres isolated from a parthenogenetic donor embryo at the morula or 4–8 cell stage were aggregated with the host blastomeres of an embryo at the synchronous or asynchronous stage.

### Evaluation of Chimeric Blastocysts by Confocal Fluorescence Microscopy

Embryos produced by the aggregation method and those produced by ICM-injection were cultured for 48 to 72 hr to examine their ability to form chimeric blastocysts. Day-6 blastocysts were observed by confocal microscopy to determine contribution of the donor cells into the ICM. Blastocysts showing fluorescent signals in the ICM were judged to be chimeric. Images of blastocysts placed in a drop of DPBS containing 5 µg/ml Hoechst 33342 in the 35-mm glass-bottom dish (Iwaki 3910-035, Asahi Techno Glass) were taken by a confocal fluorescence microscope (FV-1000, Olympus, Tokyo, Japan) with 10-µm optical sections.

### Generation of Chimeric Fetuses

To test whether the blastocysts generated by the aggregation method can give rise to chimeric fetuses, embryo transfer experiments were conducted (Figure 1). Donor ICMs derived from IVF blastocysts were aggregated with host blastomeres isolated from two parthenogenetic embryos at the morula or 4–8 cell stage. Aggregated embryos were cultured for 1 to 2 days, and blastocysts obtained were transferred to recipient gilts. Pregnant recipients were laparotomized to recover somite stage fetuses at day 18 of gestation.

Blastocysts (day 5 and 6) obtained by aggregation of two parthenogenetic morulae without donor ICMs were also transferred to a recipient to verify the developmental ability of the host embryos.

Crossbred (Large White/Landrace × Duroc) prepubertal gilts, weighing between 100 and 105 kg, were used as the recipients of the chimeric blastocysts. The gilts were given a single intramuscular (i.m.) injection of 1,000 IU eCG (ASKA Pharmaceutical) to induce estrus. Ovulation was induced by an i.m. injection of 1,500 IU hCG (Kyoritsu Seiyaku Corporation, Tokyo, Japan), which was given 66 hr after the injection of eCG. The blastocysts were surgically transferred into the uterine horns of the recipients approximately 146 hr after hCG injection.

### Evaluation of Chimerism in the Fetuses

The recipient gilts were autopsied on day 18 of gestation to collect somite stage fetuses. Fetuses showing the huKO-fluorescence in fluorescence microscopy (MVX10, Olympus) were evaluated to be chimeric. Chimerism was verified by PCR amplifying the sequences of huKO transgene in the genomic DNA extracted from the fetuses using DNeasy Blood and Tissue Kit (QIAGEN, Inc., Hilden, Germany).

Fetuses were also analyzed by genotyping to detect female/male chimerism. In the present study, chimeric embryos were composed of parthenogenetic host embryos; hence, the sex chromosome composition of those embryos was XX. However, half of the donor ICMs obtained from the IVF blastocysts theoretically had XY-chromosome composition. Therefore, detection of Y chromosome-specific sequences [40] in the fetuses’ genomic DNA was used to determine the chimerism. Nested PCR for detection of huKO transgene was performed using following primers: 5′- AGCACGAAGTCTGGAGACCTCTG-3′ and 5′- AGGTGGTCTTGAACTGGCACTTGTG-3′ for the first round of PCR; 5′- ACCTTACACAGTCCTGCAGACC-3′ and 5′- GCCAGCTTCAGGAACATGGT-3′ for the second round of PCR. The cycle conditions of both PCR were 95°C for 1 min, followed by 95°C for 30 sec, 68°C for 30 sec, and 72°C for 1 min per cycle for 25 cycles. The primers used to amplify the porcine male-specific sequences for sex determination were 5′- AAGTGGTCAGCGTGTCCATA-3′ and 5′- TTTCTCCTGTATCCTCCTGC-3′ [40]. The cycle conditions were 95°C for 1 min, followed by 95°C for 30 sec, 58°C for 5 sec, and 72°C for 20 sec per cycle for 25 cycles.

To quantify chimerism of the fetuses, sagittal sections of the huKO-positive fetuses were analyzed after immunostaining with polyclonal antibody against huKO (MBL Co., Ltd., Nagoya, Japan). The chimeric fetuses with fluorescence were fixed with 4% (w/v) paraformaldehyde, embedded in paraffin blocks and thin-sectioned. Paraffin-embedded sections were deparaffinized with xylene and hydrated with graded ethanols. Each section was incubated with anti-huKO antibody (1∶100) for 0.5 hr at room temperature. Distribution of huKO expressing cells in each section was determined by peroxidase staining using Histofine® kit (Nichirei Biosciences, Inc., Tokyo, Japan) with haematoxylin counterstaining. Proportion of the huKO-positive cells in the sections covering entire body of the fetuses was measured by image analysis software (ImageJ, http://rsb.info.nih.gov/ij/).

### Statistics Analysis

Statistical analyses were performed using the SPSS 16.0 software (SPSS, Inc., Chicago, IL, USA). Differences between two groups were analyzed using the χ^2^-test. For comparisons among three groups or more, the data were subjected to arcsine transformation and evaluated by one-way analysis of variance (ANOVA) followed by multiple comparisons by Tukey’s test. The level of significance was set at *P*<0.05.

## Results and Discussion

### Aggregation of Donor ICMs and Parthenogenetic Blastomeres to Produce Chimeric Blastocysts

Our first step was to address the question of whether chimeric blastocysts could be produced efficiently *in vitro* using ICMs as the donor cells for aggregation with parthenogenetic embryos. At the same time, we sought to determine whether the developmental stage of the host embryos influenced the production rate of chimeras (Figure 1).

Intact ICMs isolated from parthenogenetic blastocysts by immunosurgery [22] were used as the donor cells. Each ICM was first stained with DiI and then placed into a microwell [41] with blastomeres disaggregated from two parthenogenetic morulae (Figure 2A). After culture, 95.8% (23/24) of the aggregates developed to form single blastocysts (Figure 2B, Table 1). Fluorescent DiI signals derived from the donor ICMs were observed in 20 of the 23 blastocysts (87.0% or 83.3% of the embryos cultured) (Figure 2C). Thus, a high rate of formation of chimeric blastocysts was achieved, which was similar to the outcome obtained when donor ICMs were injected into parthenogenetic morulae (Figure 2G–I, Table 1).

**Table 1:** *In vitro* development of the chimeric embryos produced by injection or aggregation method.

Donor cells	Method	Stage of host embryos	No. of embryos cultured	Blastocysts (%)	Chimeric blastocysts (%)
ICM	Injection	Mourla	29	27 (93.1)	24 (82.8)
ICM	Aggregation	Morula**	24	23 (95.8)	20 (83.3)
ICM		4–8 cell**	26	23 (88.5)	17 (65.4)
Morula*		Morula*	34	31 (91.2)	24 (70.6)
Morula*		4–8 cell*	26	23 (88.5)	17 (65.4)
4–8 cell*		4–8 cell*	46	37 (80.4)	27 (58.7)

* Blastomeres isolated from single embryos.

** Blastomeres isolated from two embryos.

We also carried out an aggregation experiment using DiI-stained ICMs and 4–8 cell-stage parthenogenetic embryos (Figure 2D). Blastocysts were formed in 88.5% (23/26) of the aggregates (Figure 2E), and 65.4% (17/26) were chimeric (Figure 2F). The rates of blastocyst and chimera formation were not significantly different from those obtained using morulae.

With these results, we have demonstrated that the efficient production of chimeric blastocysts can be achieved by an aggregation method using donor ICMs and host parthenogenetic embryos. The developmental stage of the host embryo does not appear to be a limiting factor, as equally efficient chimera production occurred with 4–8 cell-stage embryos (day 3), which are more distant in developmental stage from the donor ICMs (day 6), compared to morula stage embryos (day 4). We believe that the ability of the aggregation method to use a wider range of host embryonic stages has practical significance.

We also confirmed that the efficiency of chimeric blastocyst production by the aggregation method using donor ICMs and host blastomeres was similar to that using aggregation of host and donor blastomeres (Table 1). The formation rates of chimeric blastocysts using ICMs (Figure 2A–C) or morula-blastomeres (Figure 3A–C) as the donor cells with morula stage host embryos were 83.3% (20/24) and 70.6% (24/34), respectively (not significantly different). Similarly, when 4–8 cell-stage embryos were used as the host, donor ICMs (Figure 2D–F) produced 65.4% (17/26) chimeric blastocysts compared to 58.7% (27/46) using synchronous (4–8 cell stage, Figure 3D–F) donor blastomeres and 65.4% (17/26) using asynchronous (morula stage, Figure 3G–I) donor blastomeres (not significantly different in either case).

In the present study, the donor ICMs were not disaggregated into single cells, but this did not appear to inhibit their incorporation by the host embryos. This suggests that iPS cells that form cellular colonies during development should also be able to aggregate with host blastomeres. However, in porcine iPS colonies with naïve type morphological characteristics, we found partially differentiated cell fractions in which *Oct3/4* promoters are highly methylated [12]. We also recently found that porcine iPS colonies with epistem cell (primed)-like characters show a reduced rate of chimeric blastocyst formation after aggregation with blastomeres (unpublished). Furthermore, Tachibana *et al*. reported that monkey blastocysts did not readily aggregate with transplanted ICMs to form chimeric embryos [42]. They identified two types of cells within monkey ICMs: a cluster of NANOG-positive epiblast cells and a covering layer of GATA-6-positive primitive endoderm cells. They suggested that cellular segregation in the ICMs of monkey blastocysts might diminish their ability to incorporate foreign cells and to develop into chimeric embryos. These findings indicate that further studies on the influence of the simultaneous presence of both partially differentiated and undifferentiated pluripotent cells in an iPS colony are necessary for investigating any possible effects on the ability to aggregate efficiently with host blastomeres to form chimeras.

In the present study, we routinely cultured donor ICMs with two parthenogenetic embryos. This protocol was adopted because use of a single morula resulted in a relatively low rate of production of chimeric blastocysts (Table 1); in many cultures, the ICM was not incorporated by the host blastomeres (data not shown). By contrast, aggregates in which the ICM was sandwiched by blastomeres from two embryos increased both the rate of blastocyst formation and the frequency of chimerism. It has been proposed previously that blastomeres located inside the morula form the ICM, while those located on the outside differentiate into trophectoderm (the “inside-outside” theory [43]). We suggest that sandwiching the donor ICM between blastomeres from two embryos is the cause of the high frequency of ICM chimerism.

As parthenogenetic embryos can be obtained easily by activating IVM oocytes, there is no increased difficulty in conducting the experiment using two host embryos per aggregation. An additional benefit is that the blastocysts generated from the blastomeres of two host embryos seem to have a high ability to develop into fetuses because of their higher cell numbers. However, the ratio of donor cells to host blastomeres may influence the rate of aggregates developing into chimeric blastocysts as well as the contribution of the donor cells to chimeric fetuses [44], [45]. Further investigation is required to determine the optimal conditions for efficient production of chimeric blastocysts and chimeric fetuses.

### Generation of Chimeric Fetuses

**Figure 4:**
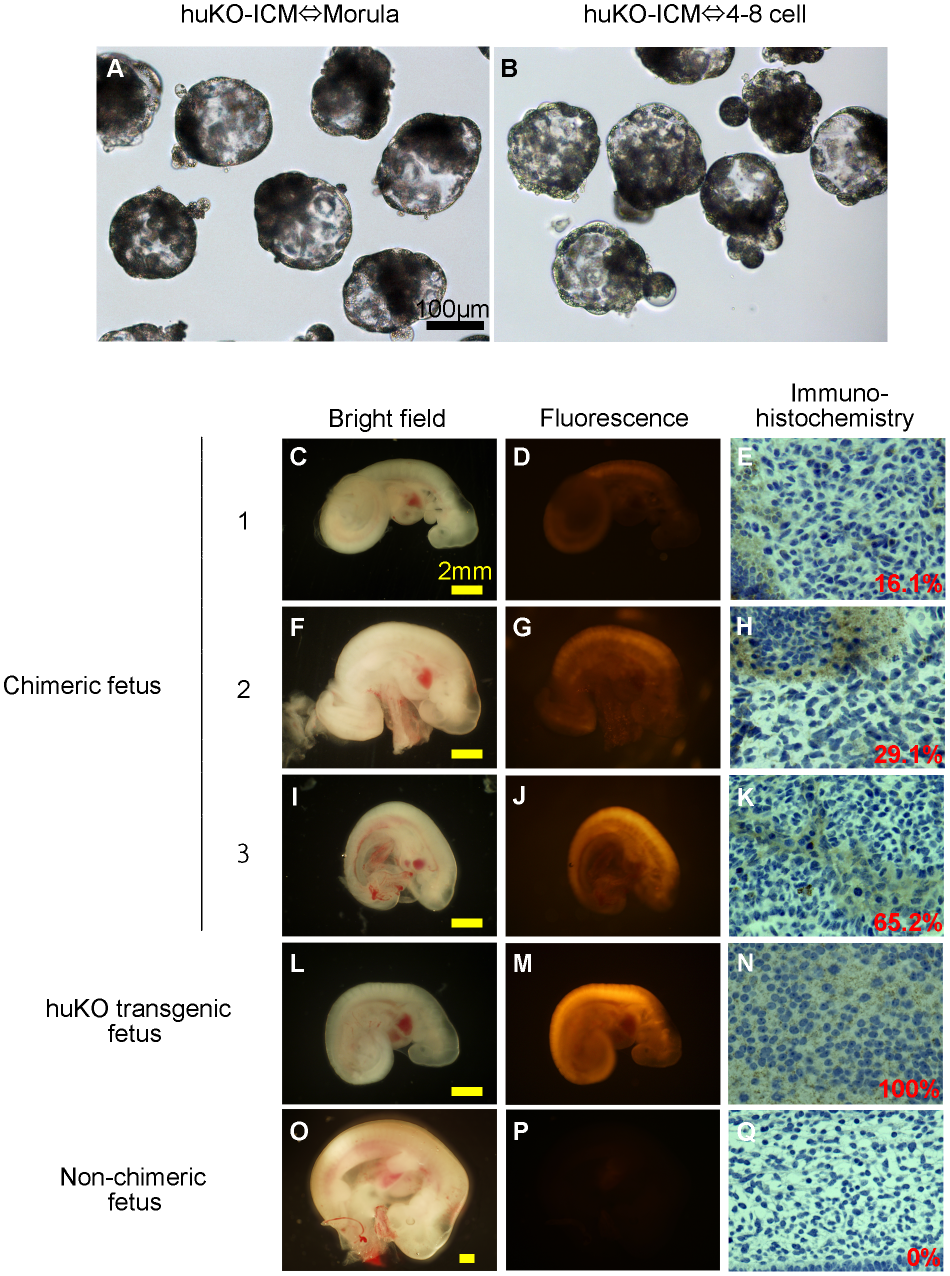
Chimeric fetuses produced by aggregation of the ICM carrying huKO transgene and parthenogenetic host embryos. (A, B) Morphological appearance of the chimeric blastocysts before embryo transfer. (C–K) Chimeric fetuses (day 18) showing huKO fluorescence derived from the donor ICM cells (C, D, F, G, I, J) and immunohistochemical images showing proportion of the donor-derived (huKO-positive) cells in the tissue of chimeric fetuses (E, H, K). (L, M, N) A day-19 fetus developed from an embryo fertilized *in vitro* with the huKO transgenic boar sperm as a positive control, showing the systemic expression of huKO (M, N). (O, P, Q) A non-chimeric fetus (day 22) developed from the aggregates of two parthenogenetic embryos as a negative control.

We carried out a transfer experiment using chimeric blastocysts to determine the contribution of donor ICM cells to chimeric fetus formation (Figure 1). In this experiment, ICMs isolated from transgenic blastocysts carrying the huKO gene were aggregated with blastomeres of parthenogenetic morulae or 4–8 cell-stage embryos. We produced 102 and 67 aggregated embryos using host embryos at the morula and 4–8 cell stages, respectively (Table 2). In total, 73 (71.6%) and 54 (80.6%) blastocysts (Figure 4A, B), respectively, developed to blastocysts and were transferred into 2 recipient gilts; all gilts were successfully impregnated.

**Table 2:** Development of the aggregated embryos into chimeric fetuses.

						Chimeric fetuses (%)		
Stage of host embryos	Aggregated embryos produced	Developed to blastocysts (%)	Blastocysts transferred	Pregnancy	Developed to fetuses (%)	huKO-positive chimera	Male-femal chimera	Total
Morula	102	73 (71.6)	73	2/2	25 (34.2)	6 (24.0)	3 (12.0)	9 (36.0)
4–8 cell	67	54 (80.6)	54	2/2	22 (40.7)	3 (13.6)	0 (0)	3 (13.6)

On day 18 of gestation, the gilts were subjected to laparotomy. In total, 25 (34.2%) fetuses were recovered from the “morula” group, while 22 (40.7%) were recovered from the “4–8 cell-stage embryo” group; the difference in the developmental rate between the groups was not significant (Table 2). A PCR analysis to detect the huKO transgene showed that 6/25 (24.0%) and 3/22 (13.6%) fetuses were chimeric (Table 2). All the PCR-positive fetuses were fluorescent due to expression of huKO, although the intensity of fluorescence varied among the fetuses indicating the presence of variation in the proportion of donor ICM derived cells among the chimeric fetuses (Figure 4). An immunostaining analysis of 6 huKO-positive fetuses showed that the proportion of donor cells in the chimeras ranged from 16 to 65% (Figure 4E, H, K). The donor cells were distributed throughout the whole body of the fetuses with no indication of any localization of huKO cells to specific organs or tissues (data not shown).

When 20 of the blastocysts developed from aggregates of two parthenogenetic morulae were transferred to a recipient, 5 (25.0%) developed to somite-stage fetuses (day 22, Figure 4O–Q), suggesting that developmental competence of the parthenogenetic host embryos were not compromised by aggregation with the donor ICMs.

The donor ICMs in this study were obtained from huKO-transgenic blastocysts produced by *in vitro* fertilization using semen of a huKO-transgenic boar that carried a single copy of the huKO gene integrated into a single chromosomal site [38]. Therefore, only half of the blastocysts obtained using the semen of this boar will be transgenic [37]. When ICMs were isolated from the blastocysts, we did not carry out any check on whether they showed huKO expression to avoid damage to the embryos by fluorescence microscopy. Thus, non-transgenic ICMs had been used as donor cells. A second factor to be considered is that half of the non-transgenic donor ICMs will be male whereas the parthenogenetic host embryos are all female. Hence, if male cells are detected in the fetuses, this is evidence of chimerism. Therefore, we screened the non-fluorescent fetuses for porcine male specific DNA sequence [40]; this analysis identified 3 fetuses in the morula group that were chimeras. Overall, therefore, we identified 9/25 chimeric fetuses in the morula group (36.0%, Table 2). No male-specific signals were detected in the huKO negative fetuses in the 4–8 cell embryo group.

The analyses described above identified chimeras based on the presence of the huKO transgene or male cells; however, other chimeras may also exist. For example, chimeras formed by the combination of a female non-fluorescent ICM and a parthenogenetic host embryo (female) would not be detectable in the present analyses. Therefore, we believe that the rate of chimeric fetuses might be greater than the detected frequency, that is, 36.0% in the morula group and 13.6% in the 4–8 cell-stage embryo group (Table 2). Regardless, we have shown here that our approach ensures that at least one fetus out of every 10 fetuses generated will be a chimera. As it is a relatively simple matter to obtain more than 10 parthenogenetic fetuses from a recipient female [32], we anticipate that one to several chimeric fetuses will be generated in a litter of fetuses when pluripotent donor cells are used. Therefore, we propose that the aggregation method using parthenogenetic host embryos is a practical approach to the evaluation of the pluripotency of iPS cells.

The aggregation method using host parthenogenetic embryos is straightforward; however, parthenogenetic embryos are expected to be restricted in their developmental capacity to the somite stage and are not expected to produce liveborn young [31], [32], [46]. In the present study, we sought to establish a method for evaluating the comparative ability of donor cells to contribute to chimeric fetuses and were not interested in the production of chimeric offspring. However, even somite stage fetuses can be valuable for tracking the differentiation of endoderm, mesoderm, and ectoderm tissues [33]. Gonadal specification can also be examined. When chimeric fetuses after the somite stage or liveborn offspring are required, it will be necessary to use IVF embryos or *in vivo*-derived embryos to provide the host cells for the aggregation method.

### Conclusion

We have demonstrated that chimeric fetuses can be produced in a highly reproducible manner by aggregation of host parthenogenetic embryos at the morula or 4–8 cell stages with donor ICM cells. To the best of our knowledge, this is the first demonstration that aggregation with parthenogenetic blastomeres is an effective means of determining pluripotency in porcine cells. The method provides a simple and highly accurate system for evaluating whether undifferentiated cells such as iPS cells possess the chimera formation ability characteristic of true pluripotency.
